# Supplementary material for: Comprehensive Annotation and Functional Exploration of MicroRNAs in Lettuce
Source: Front Plant Sci. 2021 Dec 24;12:781836. doi: 10.3389/fpls.2021.781836 (PMC8739914; doi:10.3389/fpls.2021.781836)
Supplement: Supplementary file 3 [file Data_Sheet_9.ZIP › GSM2230752.plot/Lsa-miRN1709_Lsat_1_v5_gn_8_132601.1_476_TPlot.pdf]

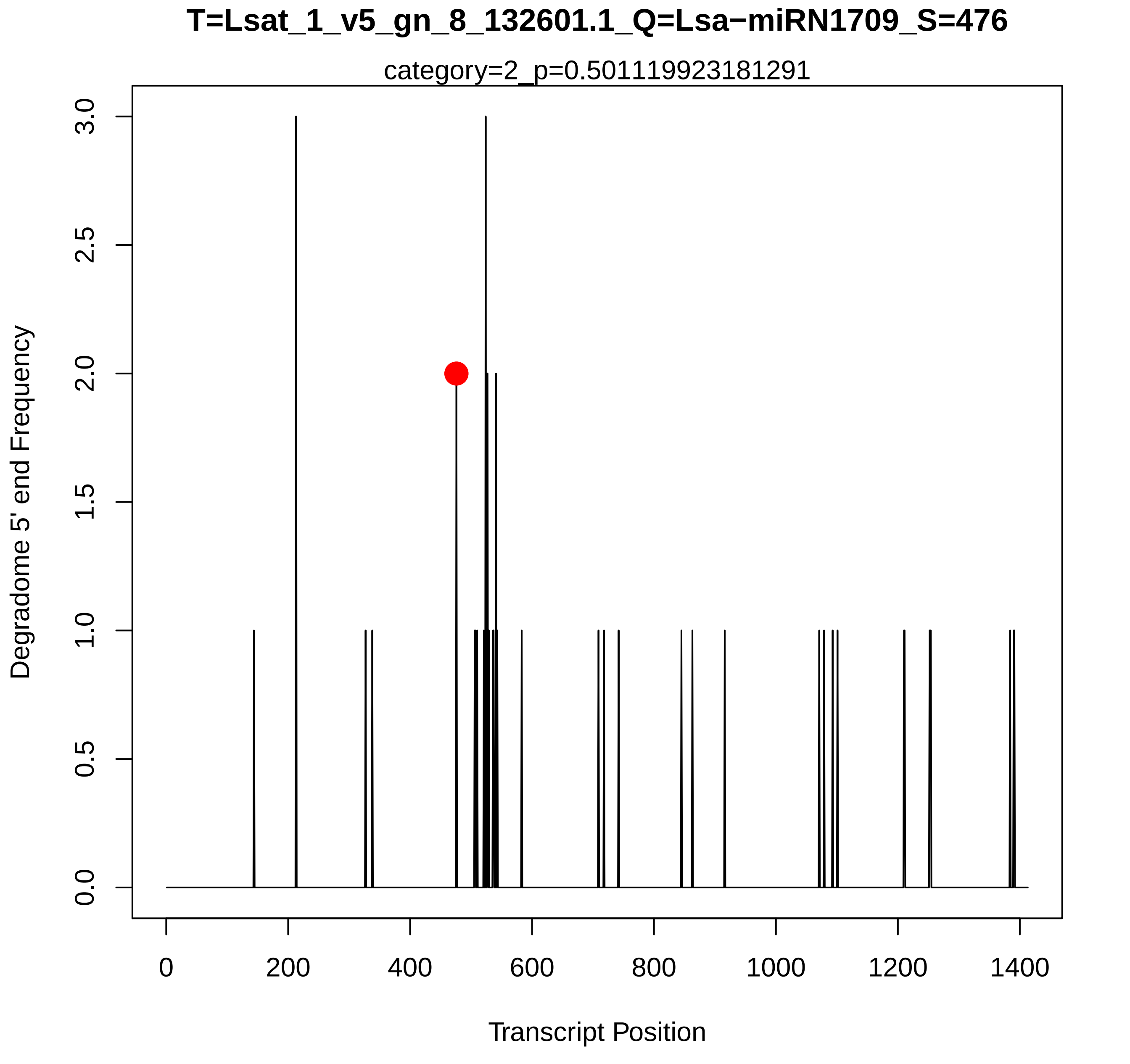
T=Lsat_1_v5_gn_8_132601.1_Q=Lsa−miRN1709_S=476
category=2_p=0.501119923181291
Degradome 5' end Frequency
Transcript Position
0.0
0.5
1.0
1.5
2.0
2.5
3.0
0
200
400
600
800
1000
1200
1400
